# Improved GSO Optimized ESN Soft-Sensor Model of Flotation Process Based on Multisource Heterogeneous Information Fusion

**DOI:** 10.1155/2014/262368

**Published:** 2014-04-10

**Authors:** Jie-sheng Wang, Shuang Han, Na-na Shen

**Affiliations:** ^1^School of Electronic and Information Engineering, University of Science & Technology Liaoning, Anshan 114044, China; ^2^National Financial Security and System Equipment Engineering Research Center, University of Science & Technology Liaoning, Anshan 114044, China

## Abstract

For predicting the key technology indicators (concentrate grade and tailings recovery rate) of flotation process, an echo state network (ESN) based fusion soft-sensor model optimized by the improved glowworm swarm optimization (GSO) algorithm is proposed. Firstly, the color feature (saturation and brightness) and texture features (angular second moment, sum entropy, inertia moment, etc.) based on grey-level co-occurrence matrix (GLCM) are adopted to describe the visual characteristics of the flotation froth image. Then the kernel principal component analysis (KPCA) method is used to reduce the dimensionality of the high-dimensional input vector composed by the flotation froth image characteristics and process datum and extracts the nonlinear principal components in order to reduce the ESN dimension and network complex. The ESN soft-sensor model of flotation process is optimized by the GSO algorithm with congestion factor. Simulation results show that the model has better generalization and prediction accuracy to meet the online soft-sensor requirements of the real-time control in the flotation process.

## 1. Introduction

Based on the differences of the surface property of solid materials, flotation process is to separate useful minerals with gangue [1], in which the economic and technical indexes (concentrate grade and flotation recovery rate) are the key controlled indicators in the production process. Their control in the flotation process is mainly according to the flotation operators' experiences by observing the states (such as the color, the size, the flow rate, texture features, etc.) of the flotation froth on the flotation cell surface to adjust the flotation tank level and change the pharmacy addition. This method of artificial observation on flotation froth has limitations of the space, time, and subjectivity, and it cannot be organically combined with computer control system to achieve high-level control [2, 3]. Inferential estimation (soft-sensor) technology can effectively solve the problem that the flotation process is difficult to online estimate the economic and technical indicators.

Domestic and foreign scholars apply digital image processing techniques to the froth feature extraction and the soft-sensor modeling of the key technical indicators in the flotation process and make a lot of achievements [4–11]. Hargrave and Hall study the diagnosis and analysis methods of the metal grade and quality and flow rate in flotation process by using the color and surface tissue. Then the statistical methods and mathematical models are utilized to find the relationship between parameters. The research results show that the color parameters of flotation froth can be used to forecast the concentrate grade in the beneficiation production process [5]. Bartolacci et al. use multivariate image analysis (MIA) and partial least squares (PLS) methods to establish the experience prediction model of flotation grade. On the other hand, the GLCM and wavelet transform analysis (WTA) methods are utilized to get the flotation froth characteristics [6]. Morar et al. utilize the machine vision method to predict the performance of the flotation process, such as concentrate grade and tailings recovery rate [7].

At home, Yang et al. aim at the question that bubble image quality is not ideal and the bubbles' size, shape, and gray scale are uneven in mineral flotation process and put forward a bubble image segmentation method based on the clustering presplit and the accuracy distance reconstruction. The size of the bubbles and other physical characteristics provide the basis for flotation control [8]. In that the multiple models additive can improve the overall prediction accuracy and the idea of robustness, Wang et al. present a multi-T-S fuzzy neural network soft-sensor model of flotation process based on the FCM clustering algorithm [9]. Yang et al. use the flotation froth video image features as auxiliary variables, establish a soft-sensor model of the flotation pulp pH value based on the sparse polynuclear least squares support vector machine (SVM), which combines the weighted local kernel function and global kernel function, and use Schmidt orthogonalization theory to reduce the multinuclear matrix [10]. Li et al. set up a soft-sensor mode by combining the principal component analysis (PCA) and extreme learning machine (ELM) methods [11].

The above established soft-sensor models of the flotation process only make use of the part of multisource heterogeneous information (real-time process datum, image feature information, and laboratory datum), not realizing information integration, coordination, and optimization of flotation process. The paper proposes ESN fusion soft-sensor method based on process datum and flotation froth image visual characteristic parameters (color features and texture features). Simulation results demonstrate the effectiveness of the proposed method.

The paper is organized as follows. In Section 2, the technique flowchart of flotation process is introduced. The ESN fusion soft sensing model of flotation process based on improved glowworm swarm optimization algorithm is presented in Section 3. In Section 4, experiment and simulation results are introduced in details. Finally, the conclusion illustrates the last part.

## 2. Technique Flowchart of Flotation Process

**Figure 1:**
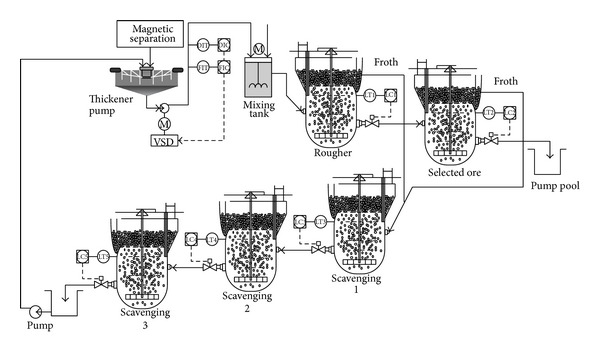
Technique flowchart of flotation process.

Flotation process is used to separate useful minerals and gangue based on the differences of the surface property of solid materials.  Figure 1 is a typical iron ore flotation process consisting of the roughing, concentration, and scavenging [11]. The system input is the fine concentrate pulp which is early output of beneficiation process in the forepart. The pulp density is about 38% and concentrate grade is about 64%. Inlet pulp is fed into the high-stirred tank through the pulp pipeline by feed pump. At the same time, the flotation reagent according to a certain concentration ratio is also fed into high-stirred tank through dosing pump. On the other hand, the pulp temperature must reach a suitable flotation temperature by heating. If the dosage is appropriate, the flotation cells can output a grade of 68.5%–69.5% concentrate.

The control objective of flotation process is to ensure the concentrate grade and the tailings recovery rate are within a certain target range. In common, based on the off-line artificial laboratory to get grade values, the operators adjust the flotation cell level and the amount of flotation reagent addition. Due to the artificial laboratory for two hours at a time, when the process variables and boundary conditions change in the flotation process, they cannot timely adjust the flotation operation variables, which results in such phenomena that the flotation concentrate grade and the tailings recovery rate are too high or too low [11]. By analyzing the flotation technique, the process variables and boundary conditions mainly include feed grade *x*_1_, feed flow rate *x*_2_, feed concentration *x*_3_, feed granularity *x*_4_, and medicament flow rate *x*_5_.

## 3. Soft-Sensor Modeling of Flotation Process

### 3.1. Structure of Soft-Sensor Model

**Figure 2:**
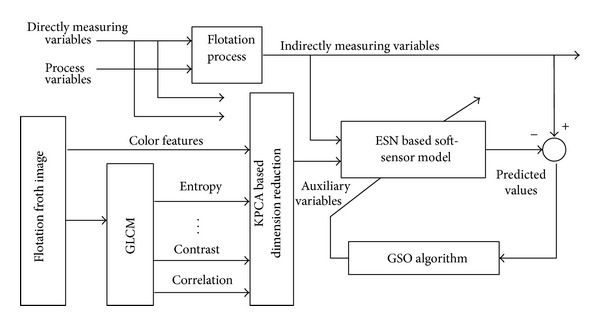
Soft-sensor model structure of flotation process.

The structure of the proposed ESN soft-sensor model optimized by the glowworm swarm optimization algorithm is shown in Figure 2 [12].

The auxiliary variables of the proposed soft-sensor model are process variables, color features, and texture features (14 texture parameters based on the calculated gray-level co-occurrence matrix by flotation froth images, such as angular second moment, contrast, correlation, sum of squares, inverse difference moment, etc.). Then KPCA method is used to realize the dimension reduction of the high-dimensional input vector composed by the normalized auxiliary variables datum in order to reduce the ESN complex. Finally, the ESN network structure parameters are optimized by the improved GSO algorithm to realize the accurate prediction of the concentrate grade and tailings recovery rate in the flotation process.

Considering a multiinput multioutput (MIMO) system, the training sample set can be expressed as *D* = {*Y*, *X*_*i*_ | *i* = 1,2 …, *m*}. *Y* is the output variable. *X*_*i*_ represents the *i*th input vector and can be expressed as *X*_*i*_ = [*x*_1*i*_,*x*_2*i*_,…,*x*_*ni*_]′ (*n* is the number of samples in the training set and *m* is the number of input variables). Soft-sensing modeling requires a datum set from the normal conditions as the modeling data. Assume that the system has *m* process variable and *n* data vectors composing the test sample datum matrix *X* ∈ *R* ^*n*×*m*^. In order to avoid the different dimension of the process variables affecting the results and obtain the easy mathematical treatment, it is necessary to normalize the datum. Set *μ* is the mean vector of *X* and *σ* is the standard deviation vector of *X*. So the normalized process variable is expressed as follows:

(1) $$\begin{array}{c} \hat{X}=\frac{X-\mu }{\sigma }. \end{array}$$

The input vector $\hat{X}$ of the training samples is fed into the ESN to obtain the predicted output $\hat{Y}$. Then the root mean square error (RMSE) is selected as the fitness of the soft-sensor model:

(2) $$\begin{array}{c} \text{RMSE}=\sqrt{\frac{\sum_{k=1}^{n}{\left({\hat{Y}}_{k}-Y_{k}^{*}\right)}^{2}}{n}}, \end{array}$$

where Y* is the actual output of training samples.

### 3.2. Extraction of Flotation Froth Color Features

Flotation froth images are obtained by the CCD camera above the flotation tank, and the computer image acquisition card converts continuous analog signal into discrete digital signals, which is conveyed into the computer for the extraction of visual characteristics of flotation froth.

**Figure 3:**
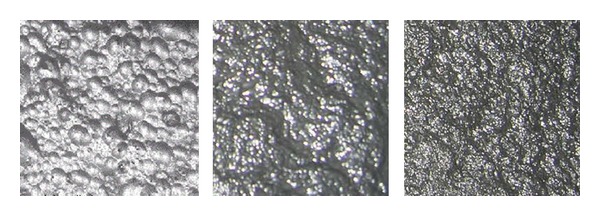
Typical iron ore flotation froth images.

Typical flotation froth image is shown in Figure 3 [13]. The froth images can be divided into three categories according to the flotation process and expert experience. (1) Bubble size is bigger, that is to say the big bubbles are mixed in the froth, the texture is shallow, texture is coarse, image complexity is small, color is pale, and SiO_2_ of froth is less. In this case, the refined iron ore grade is low. (2) Bubble size is appropriate, more uniform, and stable, color is gray, the texture is fine, and the image is more complex. At this time, the flotation process is stable and the refined iron ore slurry grade meets the requirements. (3) Froth color becomes darker, even partial black, froth is finer, and even some bubbles are difficult to distinguish, and texture is very complicated. At this case, the SiO_2_ content of froth is higher, although the iron concentrate grade is higher and the pharmaceutical is added excessively, which does not meet the economic requirements.

Flotation operators are mainly based on the color and gray closeness of the flotation froth, the luminance information of the froth surface, and the measured process variables to realize the real-time optimal control of the flotation process. Therefore, the froth color (or light intensity) reflects the information of the minerals concentration in the surface froth. The collected images are *RGB* true color images, which adopt the red, green, and blue three components. But they are often closely related. In addition, the color information of hue, saturation, and intensity (*HIS* model) is relatively close to the people color visual perception. In *HIS* model, *H* is hue representing the different colors such as red, green, and blue; *S* is saturation representing the color, such as magenta, red; *I* is the brightness indicating the brightness level of the color. In industrial applications, the range of *S* is [0, 1] corresponding from unsaturated to fully saturated state (without any white). The range of *I* is [0, 1] corresponding from dark to light color. The conversion equations from *RGB* to *HIS* are expressed as follows:

(3) $$\begin{array}{c} I=\frac{R+G+B}{3},\\ S=1-\frac{3}{R+G+B}\left[\min\left(R,G,B\right)\right]. \end{array}$$

Thus, the saturation (*S*) and brightness (*I*) of the flotation froth images have got to be applied in representing the relationship between the concentrate grade, the tailings recovery rate, and the color characteristics of flotation froth images.

### 3.3. Extraction of Flotation Froth Texture Features Based on GLCM

**Figure 4:**
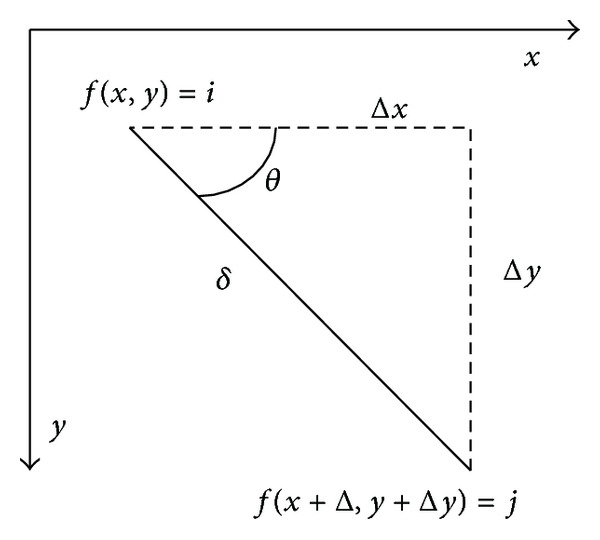
Grey-level co-occurrence matrix.

The texture statistical characteristics of the flotation froth image can reflect the working conditions of the flotation process. Image texture is formed by different gray values distributed in the space position and repeated alternate changes; thus two pixels will exist in a certain gradation relationship, which is known as the correlation characteristics of the gray space. Gray-level co-occurrence matrix (GLCM) is an important method used to analyze the image texture features, which is based on the second combination condition probability density function of estimated image [14].  Figure 4 is a GLCM schematic diagram, wherein *i* and *j* denote the gray scale value of the corresponding pixel.

The GLCM means a kind of statistical form of the joint distribution of two pixels, that is to say, the simultaneous occurrence probability *P*(*i*, *j*, *δ*, *θ*) of two pixels. They are the pixel with gray scale *i* from the image*f*(*x*, *y*) and the pixel (*x* + Δ*x*, *y* + Δ*y*) with gray scale *j* at declination *θ* and distance *δ*. The mathematical formula is expressed as follows:

(4) P(i,j,δ,θ)={[(x,y),(x+Δx,y+Δy)] ∣ f(x,y)=i, f(x+Δx,y+Δy)=j; x=0,1,…,Nx−1; y=0,1,…,Ny−1},

where *i*, *j* = 0,1,…, *L* − 1, *x* and *y* are the coordinates of the image pixel, and *L* is the image gray level.

According to the above definitions, the element in *i*th row and *j*th column of the constituted GLCM represents the appearance frequency of all pixels with the *i* and *j* gray level in the *θ* direction and *δ* length. GLCM has rich characteristics parameters describing the image textures with different angles. Haralick et al. [15] once proposed 14 GLCM based texture parameters, whose calculation formulas are shown in Table 1.

### 3.4. KPCA Based Dimension Reduction of Soft-Sensor Model

The visual characteristic parameters (2 color features and 14 texture features) of the flotation froth images and 5 process variables are served as the input variables of the ESN fusion soft-sensor model to predict the concentrate grade and flotation recovery rate. A batch of flotation froth images and the measured values of the process variables in corresponding period are collected to establish the soft-sensor model. The input-output samples are shown in Table 2.

The flotation froth image characteristics and process variables and boundary conditions are selected as the auxiliary variables of the proposed soft-sensor model to realize the integration of multisource heterogeneous information in the flotation process. But there are the problems of the jumbled information and repeated expression. If the input vector dimension of the ESN model is too long, the network topology will be complex and training will become very complex. Therefore, the kernel principal component analysis (KPCA) method [16] is adopted to reduce the model dimension of the ESN soft-sensor model.

KPCA is a nonlinear promotion of introducing the concept of the kernel function into the principal component analysis (PCA) method, which has better ability to handle nonlinear problems than PCA. Its basic principle is described as follows [17].

Given sample set *x*_*i*_ (*i* = 1,2,…, *M*) and *x*_*i*_ ∈ *R* ^*N*^, the nonlinear mapping relation is given as follows:

(5) $$\begin{array}{c} \phi :R^{N}⟶F,\;\;x⟼\phi \left(x\right). \end{array}$$

So the sample *x*_*i*_ is mapped as *ϕ*(*x*_*i*_). Then the covariance matrix of new sample space is calculated according to the following equation:

(6) $$\begin{array}{c} R=\frac{1}{M}\overset{M}{\underset{i=1}{\sum }}\phi \left(x_{i}\right){\left(x_{i}\right)}^{T}. \end{array}$$

The eigenvalue decomposition is carried out according to the following equation:

(7) $$\begin{array}{c} \lambda Q=RQ, \end{array}$$

where *λ* (*λ* > 0) is the eigenvalue of *R* and *Q* is the corresponding eigenvector. By multiplying *ϕ*(*x*_*i*_) on both sides of (7), we obtain the following:

(8) $$\begin{array}{c} \lambda \left(\phi \left(x_{i}\right)\cdot Q\right)=\left(\phi \left(x_{i}\right)\cdot RQ\right),\;i=1,2,\ldots ,M. \end{array}$$

And coefficient *α*_*i*_ (*i* = 1,2,…, *M*) exists to make the following equation:

(9) $$\begin{array}{c} Q=\overset{M}{\underset{i=1}{\sum }}\alpha_{i}\phi \left(x_{i}\right). \end{array}$$

By combining the above two equations, matrix *K*(*M* × *M*) is defined as follows:

(10) λ∑i=1Mαi(ϕ(xk),ϕ(xi)) =1M∑i=1Mαi(ϕ(xk),∑j=1Mϕ(xj))(ϕ(xj),ϕ(xi)),Ki,j=(ϕ(xi)ϕ(xj))=K(xi,xj).

Set *α* is the corresponding eigenvector of the kernel matrix *K*. Then, consider the following:

(11) $$\begin{array}{c} K\alpha =M\lambda \alpha , \end{array}$$

where *α* = (*α*_1_,*α*_2_,…,*α*_*M*_)^*T*^.

Assume that the solution of (11) is *λ*_1_ ≥ *λ*_2_ ≥ ⋯≥*λ*_*P*_ ≥ ⋯≥*λ*_*M*_. *λ*_*P*_ is the last nonzero eigenvalue, whose corresponding eigenvector is *α*_1_^*k*^,…, *α*_*p*_^*k*^,…*α*_*M*_^*k*^. Then the eigenvector of *F* is normalized according to the following equation:

(12) $$\begin{array}{c} \left(Q^{k}\cdot Q^{k}\right)=I,\;k=1,2,\ldots ,p. \end{array}$$

Put *Q* = ∑_*i*=1_^*M*^ *αϕ*(*x*_*i*_) and *K*_*ij*_ = (*ϕ*(*x*_*i*_)*ϕ*(*x*_*j*_)) into (12) to obtain the following:

(13) I=∑i,j=1Mαikαjk(ϕ(xi)ϕ(xj))=∑i.j=1MαikαjkKij=αkKαk=λk(αk·αk), k=1,2,…,p.

The principal component of a new sample *x*_*i*_ is obtained by projecting mapping sample *ϕ*(*x*) of *F* into *Q* ^*k*^, which is described in the following equation:

(14) $$\begin{array}{c} \left(Q^{k}\cdot \phi \left(x\right)\right)=\overset{M}{\underset{j=1}{\sum }}\alpha_{j}^{k}\left(\phi \left(x_{i}\right)\phi \left(x\right)\right)=\overset{M}{\underset{j=1}{\sum }}\alpha_{j}^{k}K\left(x_{j},x\right). \end{array}$$

For the sake of simplicity, $\hat{K}=K-I_{M}K-KI_{M}+I_{M}KI_{M}$ is used to substitute kernel matrix of all mapping samples, among which (*I*_*M*_)_*ij*_ = 1/*M*. The paper adopts the Gaussian function as the KPCA kernel function, which is described as follows:

(15) $$\begin{array}{c} K\left(x_{j},x\right)=\exp\left\{-\frac{{\left|x_{j}-x\right|}^{2}}{\sigma^{2}}\right\}. \end{array}$$

Based on the above mentioned principles, the procedure of KPCA algorithm is described as follows.

 Calculate kernel matrix $\hat{K}$;
 calculate eigenvalues and eigenvectors of kernel matrix $\hat{K}$;
 sort eigenvalues with the descend order. Assume that *λ*_1_ ≥ *λ*_2_ ≥ ⋯≥*λ*_*M*_. Calculate the contribution ratio by (16) to decide the number of the extracted character information (*p*). Consider the following:
  (16) $$\begin{array}{c} \varphi \left(p\right)=\frac{\sum_{i=1}^{p}\lambda_{i}}{\sum_{i=1}^{M}\lambda_{i}}. \end{array}$$
(1) The eigenvectors in accordance with the previous *p* (1 ≤ *p* ≤ *M*) biggest eigenvalues are normalized according to (13).
(2) Calculate a new principal component according to (14).

The historical datum of input variables in the soft-sensor model is carried out by kernel principal component analysis, whose results are described in the Table 3. It can be seen that the contribution ratio of the previous 5 principal components has already exceeded 90%. Thus, the principal components obtained by the KPCA on the original variables datum are the input of the ESN, which not only reserved the character information of original variables but also simplified the structure of ESN.

### 3.5. Echo State Network

**Figure 5:**
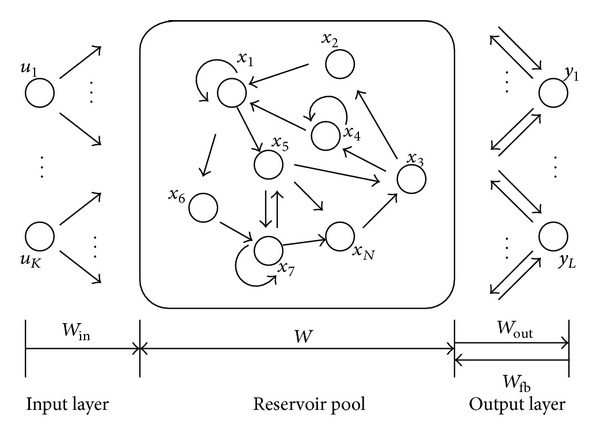
Diagram of an echo state network.

Echo state network (ESN) is a new type of recurrent neural network proposed by Jaeger [18–20]. Its internal dynamic reserve (Dynamic Reservoir, DR) pool has a large number of sparse connected neural units, which contain the operational status of the system and have the short-term memory function (the ESN echo effect). The echo effect makes the network realize the approximation effect on the learning system. A typical ESN structure is shown in Figure 5. Its basic equations can be represented as follows:

(17) $$\begin{array}{c} x\left(k+1\right)=f\left(W^{\text{in}}u\left(k+1\right)+Wx\left(k\right)+W^{\text{fb}}y\left(k\right)\right),\\ y\left(k+1\right)=f^{\text{out}}\left(W^{\text{out}}\left(u\left(k+1\right),x\left(k+1\right),y\left(k\right)\right)\right), \end{array}$$

where *f* is the DR internal activation function, usually using the Sigmoid type function to make the ESN have good nonlinear characteristic; *x*(*k*) is the DR state variable on *k* time; *u*(*k*) is the system input vector on *k* time; *y*(*k*) is the network output; *W* ^in^(*N* × *K*) is the input weight matrix; *W*(*N* × *N*) is the connection matrix among the DR internal neurons, which usually keeps the sparse connection of 1%~5% and the spectral radius less than 1 in order to make the DR have dynamic memory ability; *W* ^fb^(*N* × *L*) is the feedback matrix between the output neurons and DR; *f* ^out^ is the activation function of the input and output units, usually using a linear function; *W* ^out^ (*L* × (*K* + *N* + *L*)) is the output weights matrix. *W* ^in^, *W*, and *W* ^fb^ are constructed before the network learning, but *W* ^out^ is calculated after learning period.

### 3.6. ESN Soft-Sensor Model Optimized by Improved GSO Algorithm

#### 3.6.1. Glowworm Swarm Optimization Algorithm

The glowworm swarm optimization (GSO) algorithm is a new swarm intelligent optimization algorithm proposed by Krishnanand et al. in 2005, which intimates the firefly's phenomena, such as self-luminous, communication, courtship and foraging, and so on. GSO has been successfully used on many fields, such as multimodal function optimization and multisource tracking and location [21, 22]. Suppose the number of fireflies is *n*, which is randomly distributed in the search space of objective function. *x*_*i*_(*t*) represents the location of the *i*th firefly, *J*(*x*_*i*_(*t*)) is the fitness function, and *l*_*i*_(*t*) is the fluorescein concentration of the *i*th firefly at the moment *t*. The movements of fireflies are updated according to the following equation:

(18) $$\begin{array}{c} l_{i}\left(t\right)=\left(1-\rho \right)l_{i}\left(t-1\right)+\gamma J\left(x_{i}\left(t\right)\right), \end{array}$$

where *ρ* ∈ (0,1) is the volatilization coefficient of fluorescein and *γ* is the enhancement factor of the volatilization coefficient. Suppose *r*_*s*_ is the perception scope of fireflies and *r*_*d*_^*i*^(*t*) is the dynamic decision range (namely, decision radius) belonging to the *i*th firefly at the moment *t*, whose upper bound of the perception scope is *r*_*s*_ (0 < *r*_*d*_^*i*^(*t*) < *r*_*s*_). So the updating formula of decision domain range is represented as

(19) $$\begin{array}{c} r_{d}^{i}\left(t+1\right)=\min\left\{r_{s},\max\left\{0,r_{d}^{i}\left(t\right)+\beta \left(n_{t}-\left|N_{i}\left(t\right)\right|\right)\right\}\right\}, \end{array}$$

where *β* is the changeable rate of field, *n*_*t*_ is the neighborhood threshold controlling the neighbor number of fireflies, and *N*_*t*_(*t*) is neighbors set of the *i*th firefly at the moment *t*. Then the formula determining the number of fireflies within the decision domain is

(20) $$\begin{array}{c} N_{t}\left(t\right)=\left\{j:||x_{j}\left(t\right)-x_{i}\left(t\right)||<r_{d}^{i}\left(t\right);l_{i}\left(t\right)<l_{j}\left(t\right)\right\}, \end{array}$$

where $||\vec{x}||$ is the norm of $\overset{⃑}{x}$.

During the movement of fireflies, the fluorescein concentration of each firefly in its neighbor set determines the moving direction. Suppose that *p*_*ij*(*t*)_ is the moving probability of the *i*th firefly moving to the *j*th firefly in the neighbor set at the moment *t*, which is calculated by the following equation:

(21) $$\begin{array}{c} p_{ij}\left(t\right)=\frac{l_{j}\left(t\right)-l_{i}\left(t\right)}{\sum_{k\in N_{i}(t)}^{}l_{k}\left(t\right)-l_{i}\left(t\right)}. \end{array}$$

Based on the moving probability *p*_*ij*(*t*)_, the roulette method is adopted to decide the moving direction of the *i*th firefly. *l*_0_ is the initial fluorescein value. Suppose the moving step *s*. Thus the following formula determines the location of the *i*th firefly at the moment *t* + 1:

(22) $$\begin{array}{c} x_{i}\left(t+1\right)=x_{i}\left(t\right)+s\left(\frac{x_{j}\left(t\right)-x_{i}\left(t\right)}{||x_{j}\left(t\right)-x_{i}\left(t\right)||}\right). \end{array}$$

#### 3.6.2. Crowded Degree Factor

**Figure 6:**
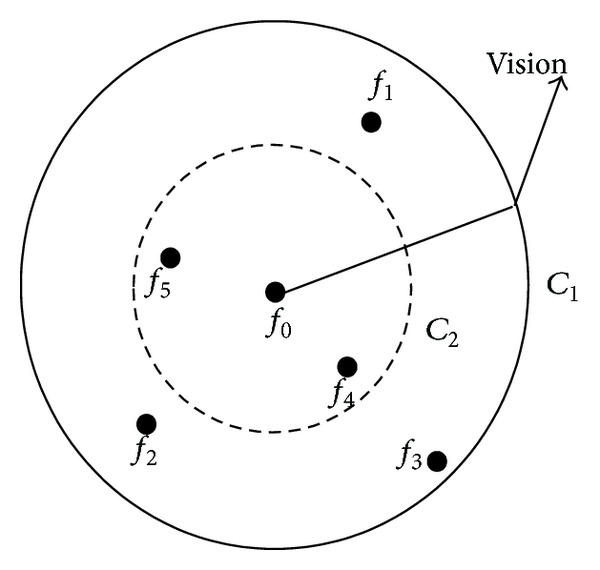
Attraction behavior description among glowworms.

The crowded degree factor *δ* is introduced to avoid the local optimum phenomena caused by the overcrowding fireflies, which can make fireflies located near the optimum point reject each other. Its principle is shown in Figure 6. For maximum optimization problem, consider the following:

(23) $$\begin{array}{c} \delta =\frac{1}{an_{\max}},\;a\in \left(0,1\right], \end{array}$$

where *a* is the close level to the optimum value and *n*_max⁡_ is the maximum number of fireflies belonging to its neighbor field. Suppose *Y*_*i*_ is the states of fireflies themselves, *Y*_*c*_ is the preceptor state value, and *n*_*f*_ is the number of partners in fireflies' neighborhood. If *Y*_*c*_/(*Y*_*i*_ *n*_*f*_) < *δ*, *Y*_*c*_ is the overcongestion state. When it comes to the minimum optimization problem, *δ* = *an*_max⁡_, *a* ∈ (0,1]. When *Y*_*c*_ *n*_*f*_/(*Y*_*i*_) > *δ*, *Y*_*c*_ is at the state of over congestion.

Unifying the crowded degree factor and the number of fireflies in their neighborhood, the behavior of the fireflies attracting each other determines its influence on the optimization results. In Figure, the firefly *f*_0_ is the best one among the fireflies *f*_1_, *f*_2_, *f*_3_, *f*_4_, and *f*_5_, whose attracting degree is *Y*_*j*_. *C*_1_ and *C*_2_ are rounds having the same center *f*_0_ and different radiuses. The closer to the round center the much greater attraction to the fireflies.

If *δn*_*f*_ ≤ 1, all fireflies are attracted to *f*_0_. If *δn*_*f*_ > 1 and *C*_2_ (attracting degree is *Y*_*j*_/*δn*_*f*_) is the attracting degrees circle, the fireflies between *C*_1_ and *C*_2_ are attracted to *f*_0_. At this moment, the larger *δn*_*f*_ is, the less the fireflies are attracted. If *n*_*f*_/*δ* ≤ 1, all fireflies in the vision are attracted to *f*_0_. If *n*_*f*_/*δ* > 1, the fireflies, whose degree is greater than *Y*_*j*_ *n*_*f*_/*δ*, are attracted to move to *f*_0_. The larger *n*_*f*_/*δ* is, the less the fireflies are attracted.

#### 3.6.3. Algorithm Procedure

The main parameters of ESN soft-sensor model are the input weight matrix *W* ^in^, the DR pool weight matrix *W*, the output feedback weight matrix *W* ^fb^, and the output weight matrix *W* ^out^. There are two kinds of cases to optimize the ESN: one is to optimize *W* ^in^,  *W*, and *W* ^fb^; the other is to optimize *W* ^out^. The paper adopts the locations of the fireflies in the improved GSO algorithm to correspond with the output connection weights matrix *W* ^out^ of ESN during the ESN training stage. Through the optimized search, the output weight matrix *W* ^out^ of ESN is trained in less samples and time. Its algorithm procedure is shown as follows.

**Step 1 (initialize the algorithm parameters)**

Initialize the parameters *W* ^in^, *W*, and *W* ^fb^ of ESN, the parameters *n*, *ρ*, *γ*, *β*, *s*, *n*_*t*_, *l*_0_, and *a* of GSO, and the maximum iteration time max *t*.

**Step 2 (initialize population)**

Aiming at the output weight matrix *W* ^out^ of ESN, randomly generate *n* fireflies to consist of the initial population *P* = {*x*_1_(*t*),…, *x*_*i*_(*t*),…, *x*_*n*_(*t*)} (*i* = 1,…, *n*). Set the iteration count value *t* = 0.

**Step 3 (calculate fitness)**

Each firefly *x*_*i*_(*t*) is set as the output weight matrix of ESN, and then the training samples are fed into the ESN soft-sensor model. The predict output is calculated by (17), and the fitness value *J*(*x*_*i*_(*t*)) is calculated by (2). In the end, (18) is used to convert the objective function values *J*(*x*_*i*_(*t*)) of firefly *x*_*i*_(*t*) into the fluorescein value *l*_*i*_(*t*).

**Step 4 (update of the individual firefly position)**

Each firefly within *r*_*d*_^*i*^(*t*) makes up its neighborhood set *N*_*t*_(*t*) (0 < *r*_*d*_^*i*^(*t*) < *r*_*s*_) according to (20) by selecting those fireflies whose fluorescein values are higher to itself, and *N*_*t*_(*t*) is regulated based on the crowded degree factor. The probability *p*_*ij*_(*t*) that the *i*th firefly moves to the *j*th firefly in its neighborhood at the moment *t* is calculated by (21). The roulette wheel method is used to select individuals to move. Then the location is updated according to (22). In the end, the dynamic decision domain radius is updated according to (19).

**Step 5 (judge the termination conditions of the proposed algorithm)**

If it meets the termination conditions (e.g., it reaches the maximum iteration number max⁡⁡*t*), the best firefly is recorded. Otherwise, *t* = *t* + 1 and go to the Step 3.

## 4. Simulation Results

With a typical flotation process as the research object, an ESN fusion soft-sensor model is established for predicting the concentrate grade and the flotation recovery rate. Firstly, the 300 input-output datum sets are determined as shown in Table 2 for training and testing the ESN soft-sensor model. Then the five nonlinear component variables obtained through KPCA dimension reduction processing process are selected as the input variables of the soft-sensor model. The former 240 samples are the training datum and the rest of the samples are used to verify the soft-sensor model's performances. The paper selects the normalized root mean square error (NRMSE), the mean square error (MSE), and the mean absolute percentage error (MAPE) as the judgment on prediction effects [17], which are defined as follows:

(24) $$\begin{array}{c} \text{NRMSE}=\sqrt{\frac{1}{T{||y_{d}||}^{2}}\overset{T}{\underset{t=1}{\sum }}{\left(y\left(t\right)-y_{d}\left(t\right)\right)}^{2}},\\ \text{MSE}=\frac{1}{T}\overset{T}{\underset{t=1}{\sum }}{\left(y\left(t\right)-y_{d}\left(t\right)\right)}^{2},\\ \text{MAPE}=\frac{100}{T}\overset{T}{\underset{t=1}{\sum }}\frac{\left|y\left(t\right)-y_{d}\left(t\right)\right|}{y_{d}\left(t\right)}, \end{array}$$

where *T* is the number of the predictive samples, *y*(*t*) is the predicted number, and *y*_*d*_(*t*) is the actual sample values.

The input dimension of ESN is 5 and the output dimension is 2. Moreover, the size of the DR pool is 100, the sparse connection rate of weight matrix of DR pool is 5%, the activation function of DR pool is tanh⁡(), and the output unit uses the linear activation function. The initial values of parameters of ESN are selected as follows: *W* ^in^ = 0.3,  *W* = 0.2, and  *W* ^fb^ = 0.03. The initial values of parameters of GSO are selected as follows: *n* = 100,  *ρ* = 0.4,  *γ* = 0.6, *β* = 0.08, *s* = 0.03,  *n*_*t*_ = 5,  *l*_0_ = 5, and  *a* = 0.2. The maximum iteration time max *t* = 500.

**Figure 7:**
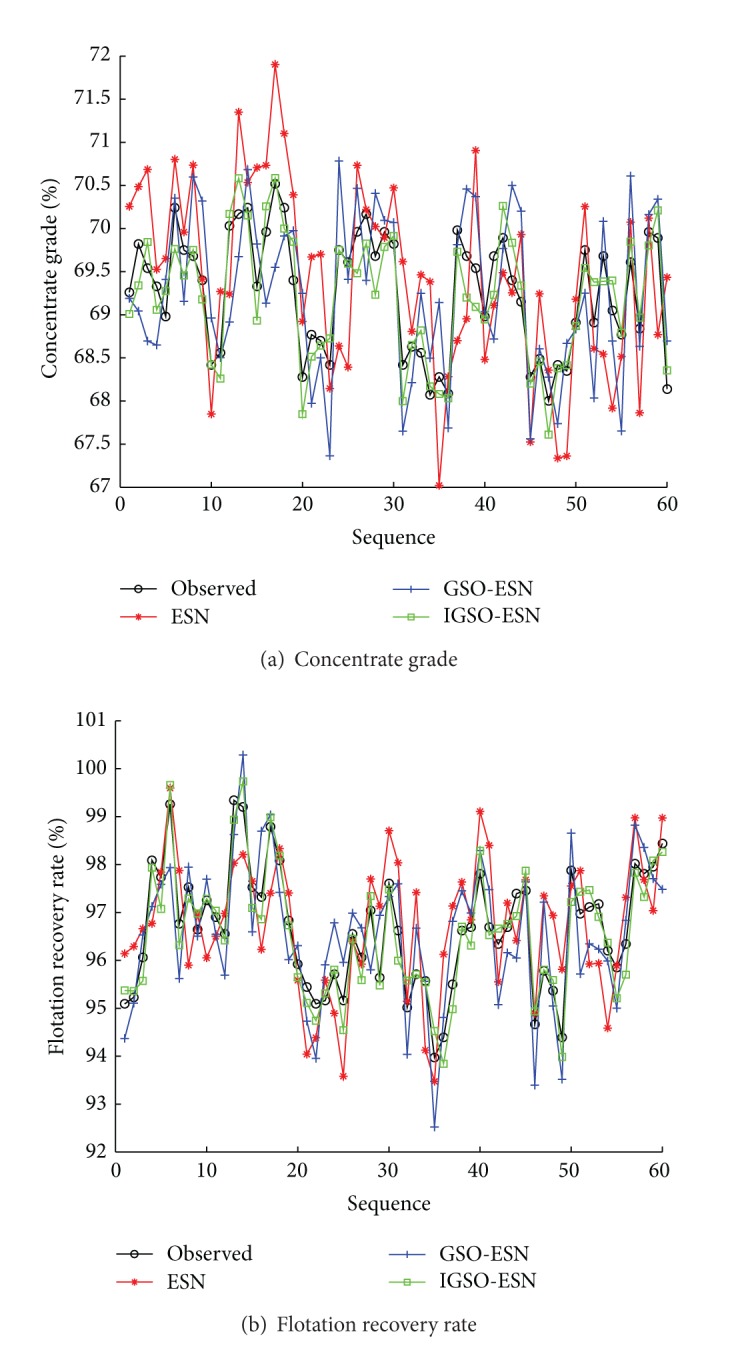
Predictive results of soft-sensor models (ESN, GSO-ESN, and IGSO-ESN).

**Figure 8:**
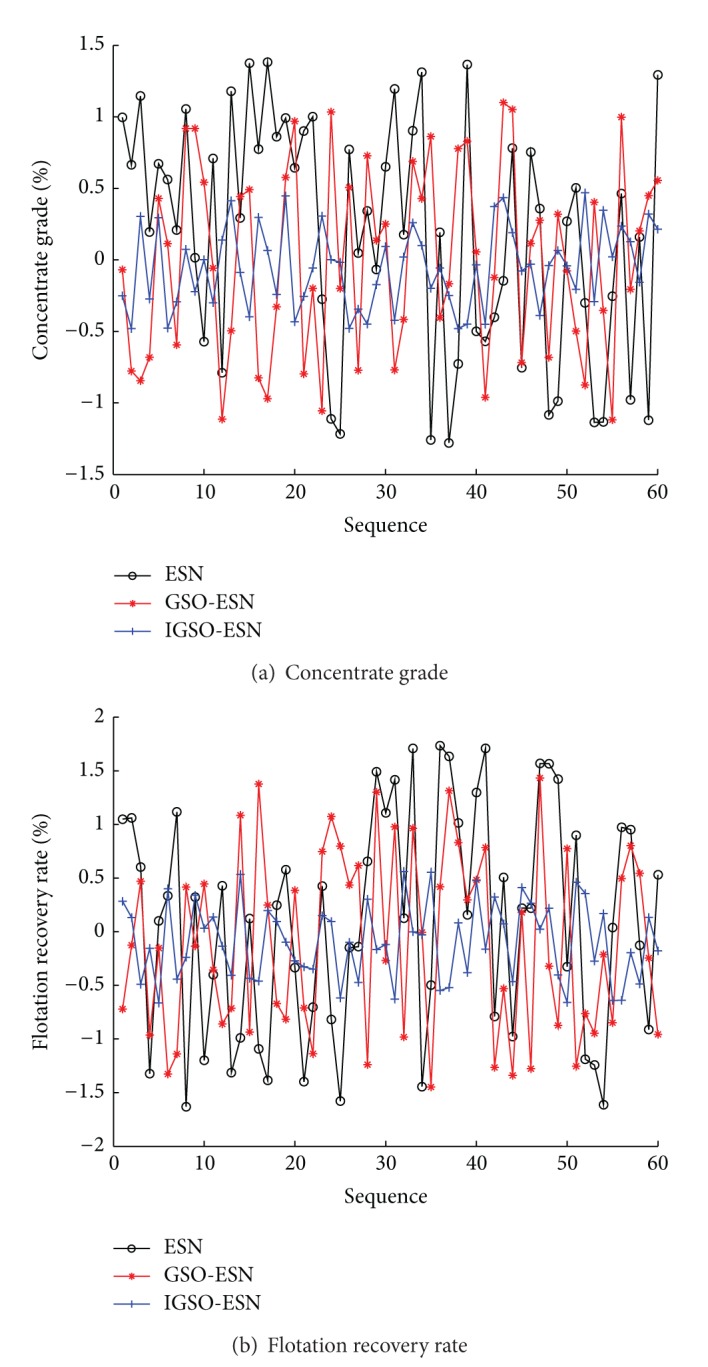
Predictive errors of soft-sensor models (ESN, GSO-ESN, and IGSO-ESN).

To illustrate the effectiveness of the proposed soft-sensor model, the improved glowworm swarm optimization (IGSO) based ESN soft-sensor model is compared with the original ESN method and the glowworm swarm optimization (IGSO) based ESN soft-sensor model. The predictive outputs and actual outputs under three methods are shown in Figure 7. The predictive error curves are shown in Figure 8. The prediction accuracies of three methods are shown in Table 4. Seen from Figures 7 and 8 and Table 4, the proposed IGSO-ESN soft-sensor model has higher predictive precision and generalization ability for the key technique index (concentrate grade and flotation recovery rate) of the flotation process than ESN soft-sensor model and GSO-ESN soft-sensor model. The proposed GSO algorithm with the crowded degree factor can adjust the structure parameters of the soft-sensor model effectively.

**Figure 9:**
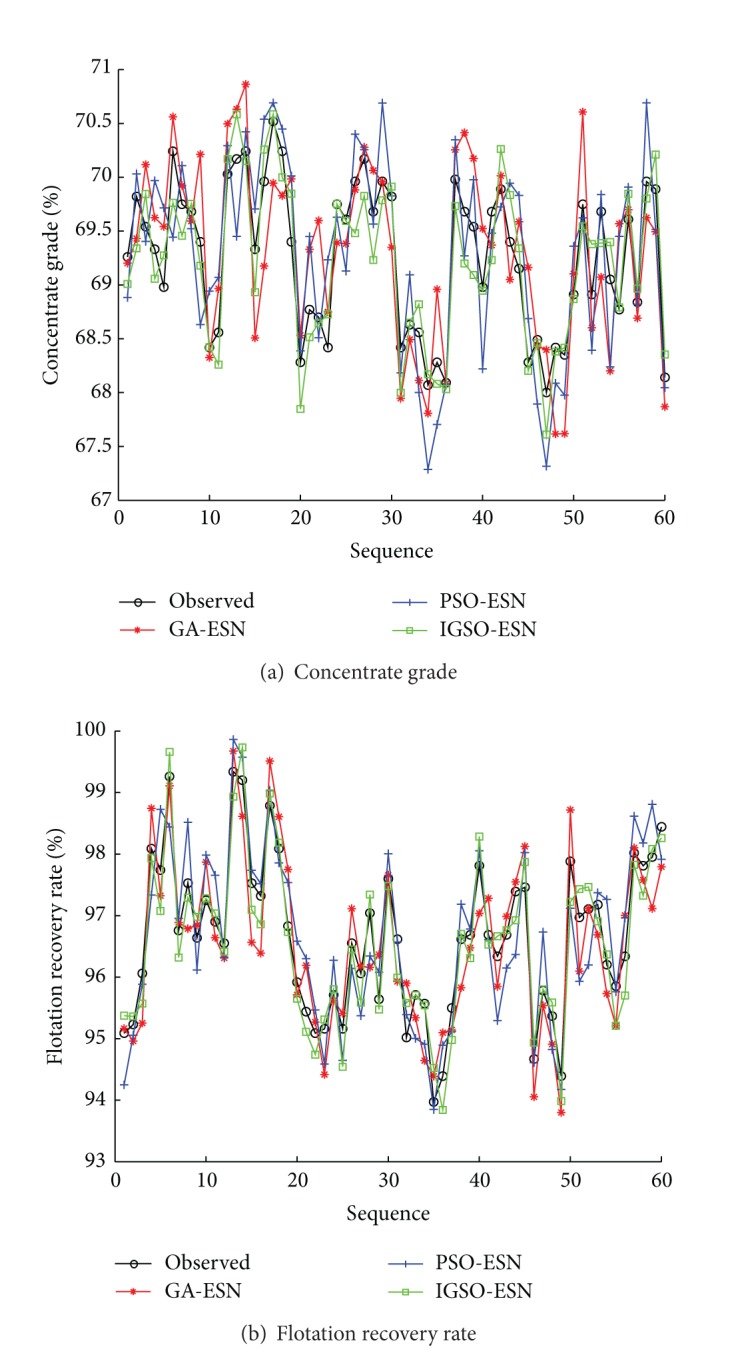
Predictive results of soft-sensor models (GA-ESN, PSO-ESN, and IGSO-ESN).

**Figure 10:**
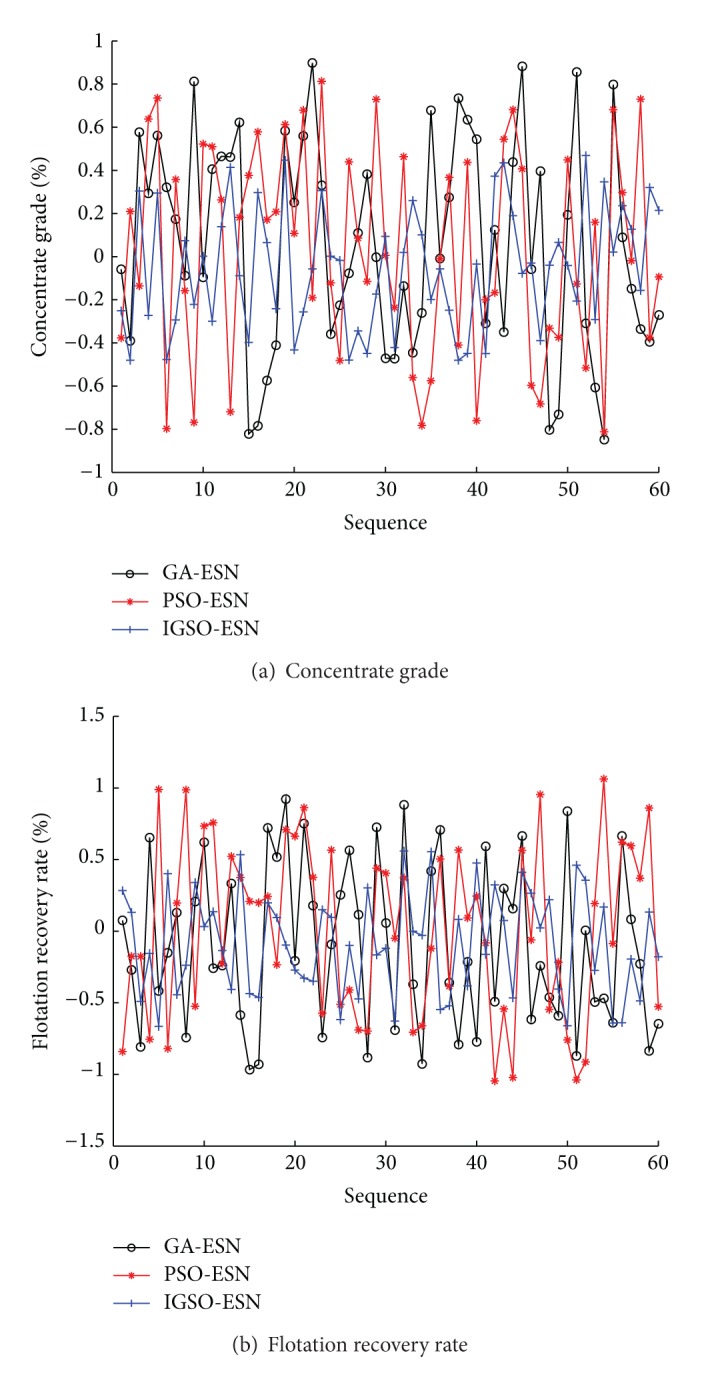
Predictive errors of soft-sensor models (GA-ESN, PSO-ESN, and IGSO-ESN).

In order to highlight the superiority of the proposed method, the comparisons have been made among IGSO-ESN soft-sensor models with two swarm intelligence based ESN soft-sensor models (GA-ESN and PSO-ESN). The predictive outputs and errors curves under three methods are shown in Figures 9 and 10. The predictive simulation has been carried out 10 times. Then the statistics analysis results of the model performances with 10 runs are listed in Table 4 based on the definition of predictive performance index.

Seen from the simulation results, the proposed IGSO-ESN predictive model has higher accuracy than the GA-ESN and PSO-ESN soft-sensor model. The successful adoption of the predictive model in the flotation process for obtaining the real-time concentrate grade and flotation recovery rate has important significance in the field of improving the production capacity and reducing production costs.

## 5. Conclusions

1. For predicting the key technical index of the flotation process (concentrate grade and tailings recovery rate), an ESN fusion soft-sensor model based on the improved GSO algorithm is proposed.
2. The fusion, coordination, and optimization of multisource heterogeneous information of flotation process are realized based on the process datum and the visual characteristic parameters of the flotation froth images (color features and texture features).
3. KPCA method is used to reduce dimension of the high dimension input variables of the soft-sensor model to extract the nonlinear principal element. Then the GSO algorithm with the crowded degree factor is used to optimize the ESN soft-sensor model.
4. The simulation results show the effectiveness of the proposed soft-sensor model for meeting the real-time monitoring requirements of the flotation process.

## Figures and Tables

**Table 1:** Grey-level co-occurrence matrix.

Texture features	Calculation equations
ASM: angular second moment	$f_{1}=\overset{L}{\underset{i=1}{\sum }}\overset{L}{\underset{j=1}{\sum }}{\left\{P(i,j)\right\}}^{2}$
Contrast	$f_{2}=\overset{L-1}{\underset{n=0}{\sum }}n^{2}\left\{\underset{\left|i-j\right|=n}{\overset{L}{\underset{i=1}{\sum }}\overset{L}{\underset{j=1}{\sum }}‍}P\left(i,j\right)\right\}$
Correlation	$f_{3}=\frac{\sum_{i=1}^{L}\sum_{j=1}^{L}\left(ij)P(i,j\right)-\mu_{x}\mu_{y}}{\sigma_{x}\sigma_{y}}$
SS: sum of squares	$f_{4}=\overset{L}{\underset{i=1}{\sum }}\overset{L}{\underset{j=1}{\sum }}{\left(i-\mu \right)}^{2}P(i,j)$
IDM: inverse difference moment	$f_{5}=\overset{L}{\underset{i=1}{\sum }}\overset{L}{\underset{j=1}{\sum }}\frac{1}{1+{\left(i-j\right)}^{2}}P(i,j)$
SA: sum average	$f_{6}=\overset{2L}{\underset{i=2}{\sum }}iP_{x+y}(i)$
SV: sum variance	$f_{7}=\overset{2L}{\underset{i=2}{\sum }}{\left(i-f_{8}\right)}^{2}P_{x+y}(i)$
SE: sum entropy	$f_{8}=-\overset{2L}{\underset{i=2}{\sum }}P_{x+y}(i)\log\left\{P_{x+y}(i)\right\}$
Entropy	$f_{9}=-\overset{L}{\underset{i=1}{\sum }}\overset{L}{\underset{j=1}{\sum }}P\left(i,j\right)\log\left\{P\left(i,j\right)\right\}$
DV: difference variance	*f*_10_ = variance of *P*_*x*−*y*_
DE: difference entropy	$f_{11}=0\overset{L-1}{\underset{i=0}{\sum }}P_{x-y}(i)\log\left\{P_{x-y}\left(i\right)\right\}$
IOC: information measures of correlation	$f_{12}=\frac{HXY-HXY1}{\max\{HX,HY\}}$
	*f*_13_ = (1 − exp⁡[−2.0(*HXY*2 − *HXY*)])^1/2^
	$HXY=-\underset{i}{\sum }\underset{j}{\sum }P(i,j)\log(P(i,j))$
	Where *HX* and *HY* are the entropy of *P*_*x*_ and *P*_*y*_.
	$HXY1=-\underset{i}{\sum }\underset{j}{\sum }P(i,j)\log(P_{x}(i)P_{y}(j))$
	$HXY2=-\underset{i}{\sum }\underset{j}{\sum }P_{x}(i)P_{y}(j)\log\{P_{x}(i)P_{y}(j)\}$
MCC: Maximal Correlation Coefficient	*f*_14_ = (second largest eigenvalue of *Q*)^1/2^ $Q(i,j)=\underset{k}{\sum }\frac{P(i,k)P(j,k)}{P_{x}(i)P_{y}(k)}$

**Table 2:** Predictive data set of the soft-sensor model.

Serial number	Color features		Texture features				Process variables and boundary conditions					Predicted variables	
	Saturation	Brightness	*f*_1_	*f*_2_	⋯	*f*_14_	Feed grade (%)	Feed flow rate (m^3^/h)	Feed concentration (%)	Feed granularity (%)	Pharmacy flow rate (L/min)	Concentrate grade (%)	Tailings recovery rate (%)
1	0.077	0.633	0.107	1.154	⋯	0.268	62.76	329	35	90	15.5	70.51	97.7
2	0.061	0.602	0.131	1.168	⋯	0.472	63.67	297	35	90	11.5	69.74	97.2
3	0.059	0.591	0.147	1.359	⋯	0.502	65.07	285	37	92	11.3	69.69	97.0
4	0.038	0.464	0.103	2.786	⋯	0.618	65.48	214	36	95	7.5	68.98	93.5
⋮	⋮	⋮	⋮	⋮	⋮	⋮	⋮	⋮	⋮	⋮	⋮	⋮	⋮
300	0.034	0.415	0.076	2.125	⋯	0.584	65.9	310	36	96	5.5	67.29	90.2

**Table 3:** Contribution rate of principle component.

Number of principal components	Percentage of variance (%)	Cumulative percentage of variance (%)
1	45.72	45.72
2	21.82	67.52
3	13.42	80.94
4	7.14	88.08
5	2.15	90.23
⋮	⋮	⋮
21	0.07	100.00

**Table 4:** Predictive error comparison of soft-sensor model.

Predicted variables	Predictive method	NRMSE	MSE	MAPE
Concentrate grade (%)	ESN	0.0122	0.6828	1.0310
	GA-ESN	0.0093	0.2379	0.6020
	PSO-ESN	0.0089	0.1918	0.5435
	GSO-ESN	0.0102	0.3562	0.7205
	IGSO-ESN	0.0069	0.0681	0.3306

Flotation recovery rate (%)	ESN	0.0096	1.0195	0.8807
	GA-ESN	0.0075	0.3593	0.5468
	PSO-ESN	0.0078	0.4158	0.5929
	GSO-ESN	0.0088	0.6626	0.7482
	IGSO-ESN	0.0060	0.1467	0.3530
